# Potential Plasma Metabolite Biomarkers of Diabetic Nephropathy: Untargeted Metabolomics Study

**DOI:** 10.3390/jpm12111889

**Published:** 2022-11-11

**Authors:** Oxana P. Trifonova, Dmitry L. Maslov, Elena E. Balashova, Steven Lichtenberg, Petr G. Lokhov

**Affiliations:** 1 Institute of Biomedical Chemistry, 10 Building 8, Pogodinskaya Street, 119121 Moscow, Russia; 2 Metabometrics, Inc., 651 N Broad Street, Suite 205 #1370, Middletown, DE 19709, USA

**Keywords:** metabolomics, mass spectrometry, blood metabolite, diagnostics, diabetic nephropathy

## Abstract

Diabetic nephropathy (DN) is one of the specific complications of diabetes mellitus and one of the leading kidney-related disorders, often requiring renal replacement therapy. Currently, the tests commonly used for the diagnosis of DN, albuminuria (AU) and glomerular filtration rate (GFR), have limited sensitivity and specificity and can usually be noted when typical morphological changes in the kidney have already been manifested. That is why the extreme urgency of the problem of early diagnosis of this disease exists. The untargeted metabolomics analysis of blood plasma samples from 80 patients with type 1 diabetes and early and late stages of DN according to GFR was performed using direct injection mass spectrometry and bioinformatics analysis for diagnosing signatures construction. Among the dysregulated metabolites, combinations of 15 compounds, including amino acids and derivatives, monosaccharides, organic acids, and uremic toxins were selected for signatures for DN diagnosis. The selected metabolite combinations have shown high performance for diagnosing of DN, especially for the late stage (up to 99%). Despite the metabolite signature determined for the early stage of DN being characterized by a diagnostic performance of 81%, these metabolites as potential biomarkers might be useful in the evaluation of treatment of the disease, especially at early stages that may reduce the risk of kidney failure development.

## 1. Introduction

Diabetic nephropathy (DN) as a specific complication of diabetes mellitus, associated with significant microvascular kidney damage, is one of the leading kidney-related disorders in patients requiring renal replacement therapy (hemodialysis, kidney transplantation), which determines the extreme urgency of the problem of early diagnosis of this disease [1,2]. Currently, the diagnosis of DN in patients with type 1 diabetes is based on an assessment of the level of albuminuria (AU) and glomerular filtration rate (GFR) [3,4,5]. However, the sensitivity and specificity of these two commonly acknowledged kidney function indices in the diagnosis of the preclinical stage of diabetic kidney damage are limited. It has been demonstrated that microalbuminuria (MAU) does not always correlate with the severity of glomerular changes, only a third of patients with MAU progress to the stage of proteinuria, and vice versa—despite the absence or regression of MAU, in some cases there may be a progressive decrease in renal filtration function [6,7]. In type 1 diabetes (T1D), the development of MAU is usually noted 5 years after the onset of the disease, while typical morphological changes in the kidney can be noted as early as 2 years after the manifestation of diabetes [8,9]. Additionally, urine albumin and serum creatinine levels are influenced by muscle mass, age, medication, and the patient’s diet, which may fail a proper renal function evaluation based on [4,10]. Thus, the search for earlier and more specific biomarkers of kidney damage in type 1 diabetes than MAU and GFR is of particular relevance for optimizing approaches to preclinical diagnosis and prevention of DN. In addition, preclinical diagnosis of renal damage will allow the initiation of renoprotective therapy at an earlier stage [11].

Metabolomics as one of the last post-genomics disciplines is also one of the most high-promising technology for researching new potential biomarkers due to its ability to study the end products of all biochemical processes in the organism (endogenous metabolites) including the environmental effect (exogenous metabolites) [12,13,14,15]. Kidneys play one of the main roles in the management of biochemical reactions in the organism and the number of metabolites excreted from the organism by directly affecting the level of circulating metabolites. Therefore, metabolites may play a functional role in the pathogenesis of kidney diseases and their complications, and the initial metabolomic changes can be detectable in DN, especially at an early stage [16,17,18]. However, using a single biomarker is often not sufficient for diagnosing complex diseases such as DN, and clinical metabolomic provides an opportunity to find a particular metabolite combination (diagnostic signature) associated with a distinct pathological state [19]. The nuclear magnetic resonance (NMR) and mass spectrometry (MS) are powerful analytical methods for providing detailed metabolomics analysis. Although each method has its own unique advantages and limitations in identifying metabolites, MS-based analytical platforms (gas chromatography (GS), liquid chromatography (LC) combined with MS, and direct injection MS) produce more comprehensive analysis [14,20,21,22,23]. That is why MS-based metabolomics analysis with bioinformatics power is particularly promising for exploring the complex pathophysiology of DN and searching for new biomarkers and signatures to diagnose early pathogenic processes leading to DN progress. If the metabolites also turn out to be causative, then they could be considered targets for drug therapy. With the aim of discovering new potential biomarkers of both early and late stages of DN and targets for drug therapy, we studied the blood plasma metabolomics profile of the individuals with T1D and DN using the direct injection mass spectrometry (DIMS) method.

## 2. Materials and Methods

### 2.1. Subjects

The study samples consisted of 80 patients with type 1 diabetes (T1D) who visited the Diabetes Institute of the National Medical Research Center for Endocrinology (Moscow, Russia) in 2014–2016 years. A cross-sectional study design with phenotypically well-defined cases and controls was chosen. The patients were divided into three specific groups. Group 1 (T1D, n = 50)–type 1 diabetes patients without diabetic nephropathy (DN) and with an estimated glomerular filtration rate (eGFR, Modification of Diet in Renal Disease equation) > 90 mL/min/1.73 m^2^, group 2 (T1D-DN1, n = 19)–type 1 diabetes patients with DN and eGFR between 60 and 89, group 3 (T1D-DN2, n = 11)–type 1 diabetes patients with DN and eGFR lower 60. The routine biochemical and blood parameters (HbA1c, fasting glucose, albumin, creatinine, urea, triglycerides, high- and low-density lipoproteins (HDL and LDL, respectively), and total cholesterol) were measured using standard automatic analyzers. The difference in baseline characteristics between the groups, including socio-demographic and anthropometric measurements, was analyzed by performing one-way ANOVA test and *p*-value < 0.05 was considered significant.

The study was approved by the relevant ethical review committee of the Diabetes Institute of the National Medical Research Center for Endocrinology (protocol №11-13 from 13 November 2013). Informed consent was obtained from all the participants at recruitment before sample collection.

### 2.2. Blood Sample Collection and Processing

Blood samples were collected in the morning after 12-h fasting. Venous blood was collected from the patients into EDTA Vacutainer plasma tubes (BD, Franklin Lakes, NJ, USA) and processed in accordance with the manufacturer’s instructions. The resultant blood plasma was frozen immediately and stored at −80 °C until further processing. The analyzed samples were subjected to one freeze/thaw cycle. The sample preparation was performed as described previously [24]. In total, 10 μL of blood plasma was mixed vigorously with 10 μL of water (LiChrosolv; Merck KGaA, Darmstadt, Germany) and 80 μL of methanol (Fluka, Munich, Germany). After incubation at room temperature for 10 min to ensure protein precipitation, the samples were centrifuged at 13,000× *g* (Centrifuge 5804R; Eppendorf AG, Hamburg, Germany) for 15 min and the supernatant was transferred to another Eppendorf™ tube. To obtain the analyzed solution each sample was diluted 50-fold by methanol with 0.1% formic acid (Sigma-Aldrich, St. Louis, MO, USA) and the internal standard (IS) Losartan (C_22_H_23_ClN_6_O, *m*/*z* = 423.169) was added to an end concentration of 10 ng/mL. All chemicals and solvents were of HPLC and UHPLC grade.

### 2.3. Mass Spectrometry Analysis

Samples were analyzed by a hybrid quadrupole time-of-flight mass spectrometer (maXis Impact, Bruker Daltonics, Billerica, MA, USA) equipped with an electrospray ionization (ESI) source. The mass spectrometer was set up and calibrated by using ES Tuning Mix (Agilent Technologies, Santa Clara, CA, USA) to detect ions with a mass-to-charge ratio (*m*/*z*) ranging from 50 to 1000 and a mass accuracy of 1–3 parts per million (ppm) in the positive ion charge detection mode. The samples were injected into the ESI source with a flow of 180 µL/h using a glass syringe (Hamilton Bonaduz AG, Bonaduz, Switzerland) and a syringe injection pump (KD Scientific, Holliston, MA, USA). All samples were analyzed in random order. The internal standard Losartan (C22H23ClN6O, *m*/*z* = 423.169) added to each sample before MS analysis was used for monitoring of matrix suppression, reproducibility, and stability of the method. Tandem mass spectrometry (MS/MS) analysis of selected precursor ions with an intensity ≥ 5000 was performed at collision energy from 10 to 40 eV.

### 2.4. Mass Spectra Processing

Mass spectra were obtained by summarizing one-minute signals using DataAnalysis version 4.1 (Bruker Daltonics). Mass spectra preprocessing including recalibration, peak detection, and peak intensity calculation were carried out by DataAnalysis software using the following parameters: peak width, 2; signal-to-noise ratio, 2; relative and absolute threshold intensity, 0.01% and 100, respectively. The resulting mass spectrometry peak masses were pooled and processed using Matlab version R2010a (MathWorks, Natick, MA, USA). The alignment of mass spectrometry data was performed as described previously [25]. If mass difference was not exceeding 0.01 Da the peaks were considered as related to the same metabolite ion. Peaks that were below the detection limit in <80% of samples in each group were removed from the analysis. Mass peak intensities were normalized as described previously [24].

### 2.5. Data Analysis and Statistics

Data in peak intensities format where samples were entered in columns, and each metabolite (variable) was added in the rows to form a matrix, which was uploaded on the MetaboAnalyst 5.0 website [26], were used. Data analysis to assess differences among the compared groups was performed on the acquired mass spectrometry metabolite profiling data using the MetaboAnalyst 5.0 through the Statistical Analysis (one factor) tool. Statistical significance was calculated by non-parametric version of one-way ANOVA (Kruskal–Wallis Test) and non-parametric Wilcoxon rank-sum test using false discovery rate (FDR) corrected *p*-values. The Wilcoxon rank-sum test (FDR adjusted *p*-value < 0.05) was used to identify each variable’s contribution to the model. The results are expressed as mean ± standard deviation (SD). To identify top pathways that are being affected by the development of diabetic nephropathy based on the putative identification of metabolites and the global interpretation of biochemical changes by the information deposited in the Kyoto Encyclopedia of Genes and Genomes (KEGG) [27] the MetaboAnalyst 5.0 through the Functional Analysis tool was used. To further find the best combination of metabolites for an accurate predictive model and evaluate their diagnostic performance by receiver operating characteristic (ROC) curve analysis (Random Forests algorithm), the MetaboAnalyst 5.0 program through the Biomarker Analysis tool was utilized. To assess the accuracy of the potential biomarkers in the models the area under the curves (AUC) and 95% confidence intervals were computed. Sensitivity, specificity, and accuracy values are calculated using formulas presented in [28]. 

### 2.6. Metabolite Annotation

Initially, the putative identification of metabolites was performed through the Functional Analysis tool of the MetaboAnalyst 5.0 using the information deposited in the Kyoto Encyclopedia of Genes and Genomes (KEGG) with mass tolerance up to 0.005 Da and allowing possible adducts in the positive ionization mode. The dysregulated metabolites were tentatively annotated via careful manual curation of all peak assignments by two orthogonal characteristics as accurate mass and isotopic distribution using the Human Metabolome Database (HMDB) [29] and METLIN [30] that meets the level 2 (putatively annotated compounds) or in some cases level 3 (putatively characterized compound classes) of metabolite identification according to the Metabolomics Standards Initiative (MSI) requirements [31]. The metabolites with sufficient mass spectrometry peak intensity were additionally confirmed by MS/MS fragmentation, and identification was based on the molecular weight, at least one specific fragment, and structure.

## 3. Results

### 3.1. Cohort Characteristics

The studied groups were classified based on the estimated glomerular filtration rate (eGFR) into T1D (type 1 diabetes (T1D) subjects without diabetic nephropathy (DN)), T1D-DN1 (T1D subjects with early stage of DN), and T1D-DN2 (T1D subjects with late stage of DN) according to the KDIGO 2012 *Clinical Practice Guideline for the Evaluation and Management of Chronic Kidney Disease* [3,5]. Albuminuria was assessed in each patient by the calculation of the albumin-to-creatinine ratio (ACR), but not used for group formation due to its low correlation with the severity of glomerular changes. The socio-demographic characteristics, anthropometric measurements, and cardiometabolic risk factors of the participants in the studied groups are summarized in Table 1. 

Analysis showed that body mass index (BMI), glycated hemoglobin (HbA1c), fasting glucose, triglycerides, high- and low-density lipoproteins (HDL and LDL, respectively), and total cholesterol were not different between the studied groups. The groups with both early and late stages of DN (T1D-DN1 and T1D-DN2, respectively) were characterized by more years since T1D was diagnosed and, as expected, had higher levels of serum creatinine and urea with a significant *p*-value < 0.001. In most patients, T1D was diagnosed during childhood or adolescence. Serum creatinine and urea levels increased with the progression of the disease and were significantly higher in T1D-DN2 than in T1D-DN1. The average age in the T1D-DN group, was significantly higher than in others. Systolic blood pressure was slightly higher in the T1D-DN2 group with a significant value of *p* = 0.029. The ACR in the control group was in the normal range, while the T1D-DN1 and T1D-DN2 groups are characterized by higher mean values but with such a high deviation that the difference between the groups is not significant.

### 3.2. Mass Spectrometry Data Analysis of Plasma in T1D Patients with or without DN

A total untargeted metabolomics analysis of the blood plasma samples using DIMS method has detected 4376 metabolite *m*/*z* features (peaks), which registered in almost all 80 samples. DIMS is a typical workflow designed for rapid untargeted analysis of the polar blood metabolome, which is able to detect amino acids, free carnitine, acylcarnitines, glycerophospholipids, lysophosphatidylcholines, phosphatidylcholines, and sphingolipids [24]. 

**Figure 1:**
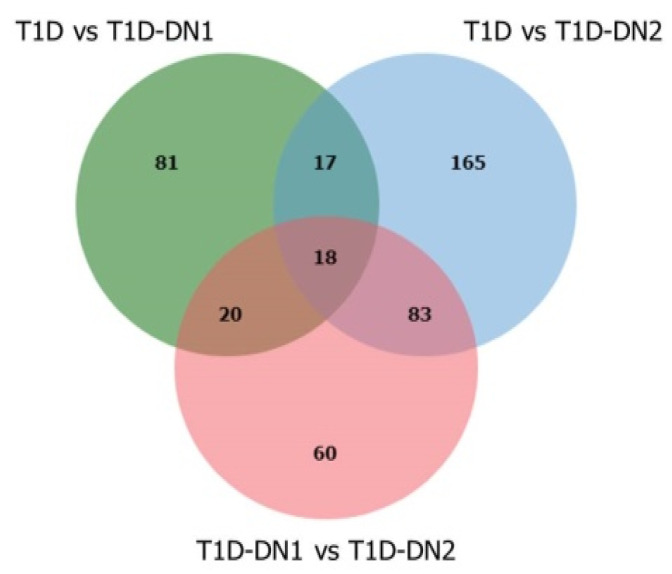
The Venn diagram of the metabolite *m*/*z* features values significantly differed between T1D and T1D-DN1 (green), between T1D-DN1 and T1D-DN2 (red), as well as between T1D and T1D-DN2 (blue).

In order to identify metabolites significantly increased or reduced in both early and late stages of DN compared to the T1D group the selection of metabolites *m*/*z* features significantly differed between T1D and T1D-DN1, between T1D-DN1 and T1D-DN2 as well as between T1D and T1D-DN2 by the pairwise Wilcoxon rank-sum test was performed (Figure 1). Among 441 significantly differed *m*/*z* features (FDR adjusted *p*-value < 0.05), only 18 significantly differed between T1D and T1D-DN1, between T1D-DN1 and T1D-DN2, as well as between T1D and T1D-DN2. A total of 136 and 283 *m*/*z* features were significantly different between T1D and T1D-DN1 and T1D-DN2, respectively, whereas 181 *m*/*z* features were different between T1D-DN1 and T1D-DN2. For further analysis, only *m*/*z* features with a *p*-value < 0.05 were selected.

### 3.3. Metabolites and Metabolite Pathways Dysregulated during DN

To identify significantly differing metabolites and discover the nature of their dysregulations associated with DN in T1D patients, the mapping of the metabolites’ *m*/*z* features with significantly altered abundance has been performed using the Functional Analysis tool in MetaboAnalyst [26]. The obtained *m*/*z* values and *p*-value were used to identify the dysregulated metabolites and associated pathways in the KEGG and HMDB databases. For the metabolites for which mass spectrometry peak intensity was sufficient, validation by MS/MS fragmentation was utilized. The annotated metabolites are listed in Supplementary Table S1. The relative abundance of the dysregulated metabolites in the blood plasma samples of the studied participants is shown as a heatmap in Supplementary Figure S1. The top metabolite pathways enriched by the significantly altered metabolites between T1D and T1D-DN1, between T1D-DN1 and T1D-DN2, as well as between T1D and T1D-DN2, are presented in Table 2. 

**Figure 2:**
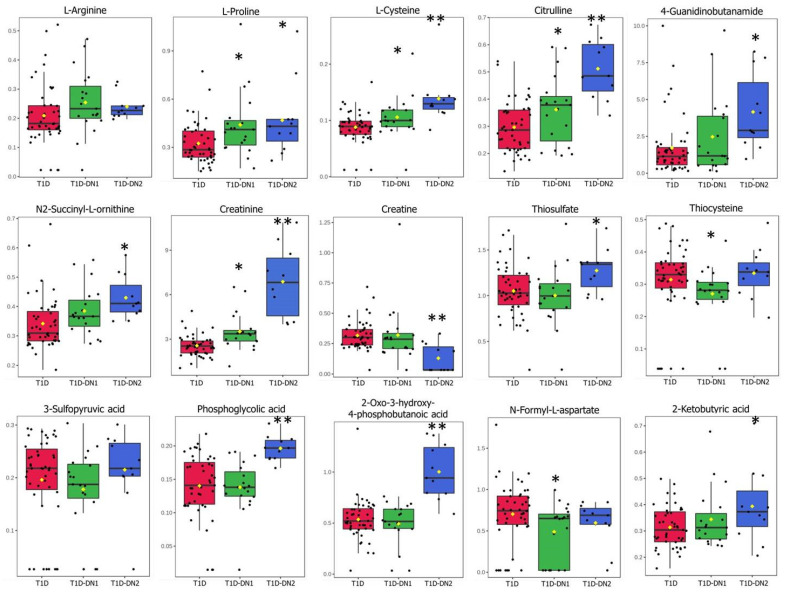
Box plots depicting abundance of the identified dysregulated metabolites in the blood plasma samples of the T1D (red), T1D-DN1 (green) and T1D-DN2 (blue) groups. Each dot represents individual blood plasma sample from each patients. *-*p*-value < 0.05; **-*p*-value < 0.01.

The majority of the emerging pathways indicated the distinct dysregulation of amino acid metabolism in DN and included aspartate and asparagine metabolism, arginine and proline metabolism, methionine and cysteine metabolism, alanine and aspartate metabolism, urea cycle/amino group metabolism and glycine, serine, alanine, and threonine metabolism (Table 2). The relative abundance of the annotated metabolites involved in the top dysregulated pathways is shown in Figure 2. The aspartate and asparagine metabolism pathways were dysregulated in both early and late stages of DN, with increased levels of L-arginine, L-proline, L-cysteine, citrulline, 4-guanidinobutanamide, and N2-succinyl-L-ornithine. However, the levels of L-cysteine, citrulline, 4-guanidinobutanamide, and N2-succinyl-L-ornithine were much higher in T1D-DN2 than in T1D-DN1 (Figure 2). At that, the urea cycle/amino group metabolism pathway was more dysregulated in the T1D-DN2 group, with significantly increased levels of creatinine and citrulline and significantly reduced levels of creatine. The difference in the levels of these metabolites between T1D and T1D-DN2 was 1.5–2.5 folds. The lower levels of thiosulfate, thiocysteine, and 3-sulfinylpyruvic acid were identified in the T1D-DN1 group which demonstrated the dysregulation in the methionine and cysteine metabolism pathway. The glycine, serine, alanine, and threonine metabolism were dysregulated in the T1D-DN2 group, with significantly increased levels of phosphoglycolic and 2-oxo-3-hydroxy-4-phosphobutanoic acids. The level of the majority of the metabolites increased/reduced gradually with the progression of the disease. While the levels of 3-sulfopyruvic acid, N-formyl-L-aspartate, thiosulfate, and thiocysteine were lower in T1D-DN1 than in T1D-DN2, and the level of L-methionine—higher in T1D-DN1 than in T1D-DN2 (Figure 2). 

Taking into account the various numbers of significant hits in Table 2, which means the number of significantly altered metabolites both up- and down-regulated between the groups, a dysregulation extent of a particular metabolite pathway may be assessed in accordance with a DN stage. For example, the glycine, serine, alanine, and threonine metabolism was characterized by more significant hits at the late stage of DN in comparison to the early stage (5 and 3, respectively). In contrast, the methionine and cysteine metabolism was characterized by more significant hits at the early stage of DN. Moreover, the number of significant hits in comparison between the T1D-DN1 and T1D-DN2 groups demonstrated the differences in the functioning of dysregulated metabolite pathways at the early and late stages of DN. Combined with the nongradual changes in the level of some metabolites, it can point to the key dysregulation of various pathways at a certain stage of the disease.

### 3.4. Metabolite Signatures for DN Diagnosis

**Figure 3:**
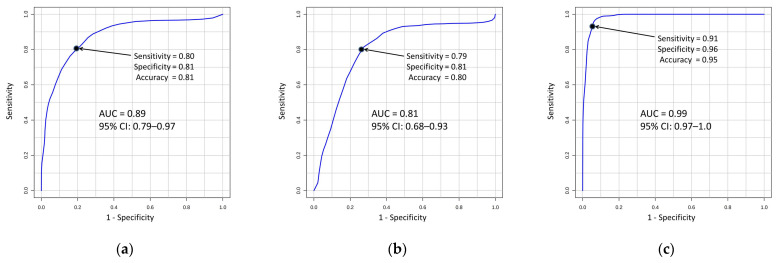
Receiver operator characteristic curves for the best combinations of metabolites for the accurate predictive models for: (**a**) both early and late stages of DN (T1D-DN1/DN2), (**b**) T1D-DN1, and (**c**) T1D-DN2. AUC–area under the curve, CI–confidence interval.

There were 17 annotated metabolites (Supplementary Table S1) with significant differences in the three comparisons (T1D vs. both early and late stages of DN (T1D-DN1/DN2), T1D vs. T1D-DN1, and T1D vs. T1D-DN2). Among these metabolites, the best combinations of metabolites (signatures) for an accurate diagnosing of both early and late stages of DN were selected according to their diagnostic effectiveness as determined by the receiver operating characteristic (ROC) curve analysis using the Biomarker Analysis tool in MetaboAnalyst. To assess the accuracy of the models, an area under the curve (AUC) with 95% confidence intervals, sensitivity, specificity, and accuracy were computed for each combination (Figure 3). 

Subsequently, 15 potential biomarkers were used for the best metabolite combinations for the diagnosing of DN cases in patients with T1D (Table 3). For the diagnosing of both early and late stages of DN (the T1D-DN1 and T1D-DN2 groups) the best combination of the mass spectrometry peaks of 11 metabolites (creatinine, L-proline, L-cysteine, creatine, 1-methylhistidine, L-arginine, citrulline, oxalosuccinic acid, N-acetyl-b-glucosaminylamine, N2-succinyl-L-ornithine, and 3-carboxy-4-methyl-5-propyl-2-furanpropionic acid) with the area under the curve (AUC) of 0.89 (sensitivity, specificity, and accuracy as 0.8, 0.81, 0.81, respectively) was determined (Figure 3a). The best combination for diagnosing the early stage of DN (the T1D-DN1 group) included 9 metabolites (creatinine, L-proline, L-cysteine, N-formyl-L-aspartate, 1-methylhistidine, oxalosuccinic acid, N-acetyl-b-glucosaminylamine, N2-succinyl-L-ornithine, and 3-carboxy-4-methyl-5-propyl-2-furanpropionic acid), and was characterized by AUC = 0.81 (sensitivity, specificity, and accuracy as 0.79, 0.81, 0.80, respectively) (Figure 3b). The best diagnostic effectiveness has been demonstrated by the combination of 12 metabolites (creatinine, L-cysteine, thiocysteine, 4-guanidinobutanamide, L-proline, creatine, 1-methylhistidine, 2-oxo-3-hydroxy-4-phosphobutanoic, citrulline, oxalosuccinic acid, N2-succinyl-L-ornithine, and 3-carboxy-4-methyl-5-propyl-2-furanpropionic acid), which could distinguish between the T1D and T1D-DN2 subjects with diagnostic score AUC = 0.99 (sensitivity, specificity, and accuracy as 0.91, 0.96, and 0.95, respectively) (Figure 3c). 

As can be seen in Table 3, creatinine, L-proline, L-cysteine, 1-methylhistidine, oxalosuccinic acid, N2-succinyl-L-ornithine, and 3-carboxy-4-methyl-5-propyl-2-furanpropionic acid were included in all combinations used for the accurate predictive models for DN regardless the disease stage. Creatinine, L-proline, L-cysteine, 1-methylhistidine, oxalosuccinic acid, N2-succinyl-L-ornithine, and 3-carboxy-4-methyl-5-propyl-2-furanpropionic acid were used in both models for diagnosing early and late stages of DN, while N-formyl-L-aspartate showed good diagnostic effectiveness for the early DN stage, and thiocysteine, 4-guanidinobutanamide, and 2-oxo-3-hydroxy-4-phosphobutanoic acid–for late DN stage only. These results again indicate the importance of various pathways in the pathogenesis of DN at different stages.

To check whether the identified dysregulations in metabolite levels are a result of the DN progression from T1D or reflect a decrease in kidney function only, we have calculated the correlation between the above-mentioned metabolites and creatinine level. In the control group (T1D patients) and in the early-stage DN patients (T1D-DN1), the correlation coefficients were quite similar (0.31 and 0.34, respectively), while in the late-stage DN patients—0.2. This indicates that dysregulation of the detected metabolites is not a direct relation to kidney function and may be a result of DN development. That is why we used the diagnostic models’ combinations with creatinine to take this indicator into account to the necessary extent. In addition, it should be noted that due to the design of the experiment, creatinine can differentiate the studied groups, but its individual diagnostic score (Supplementary Table S1) is lower than any of our combinations. Thus, all additional metabolites enhanced the diagnostic characteristics of the combinations (signatures).

## 4. Discussion

We have analyzed the untargeted blood plasma metabolomics profiles associated with both early and late stages of diabetic nephropathy (DN) in patients with type 1 diabetes (T1D) using the DIMS method. DIMS is a widely used approach for fast and sensitive analysis of sample-specific metabolite ions distribution. This method and liquid chromatography-mass spectrometry (LC-MS) have their limitations, but in contrast to LC-MS-based standard practice, DIMS is not time-consuming and more suitable for high-throughput analysis [20,22,23,24,32]. Currently, the diagnosis of DN in patients with T1D is based on an assessment of the level of albuminuria (AU) and glomerular filtration rate (GFR), when morphological changes in the kidney are manifested clinically [4,8]. In our study, the studied groups were classified based on the estimated GFR according to the KDIGO 2012 *Clinical Practice Guideline for the Evaluation and Management of Chronic Kidney Disease* [3,5]. AU was assessed in each patient by calculation of the albumin-to-creatinine ratio (ACR), but was not used for group formation due to its low correlation with the severity of glomerular changes and DN stage (Table 1) [6,7]. It was shown that in T1D, the development of microalbuminuria (MAU) is usually noted 5 years after the onset of the disease, while typical morphological changes in the kidney can be noted earlier [8]. That is why the studied subjects with both early and late stages of DN were characterized by more years since T1D was diagnosed and had higher levels of serum creatinine and urea than the T1D patients who had not progressed to DN. While the early diagnostics would provide an opportunity to delay or prevent the disease’s development through on-time therapy. 

The important findings of the study are the metabolites and associated pathways that are dysregulated in both early and late stages of DN and the potential metabolite signatures for diagnosing DN in patients with T1D. Statistical analysis of the metabolomics data coupled with functional analysis based on system biology knowledge allowed us to identify the involvement of some amino acids metabolism and urea cycle in the pathogenesis and progression of DN. In an attempt to identify metabolite signatures for detection, both early and late stages of DN 15 metabolites were identified as potential biomarkers in this study. The fact that most of the revealed dysregulated metabolites are amino acids and derivatives, and that the top metabolic pathways are directly related to amino acid metabolism points to the importance of these biochemical processes in the pathogenesis and progression of DN [17,18,33,34,35]. 

As expected, creatinine was found in the study with an up-regulated profile in the DN subjects and was much higher in T1D-DN2 than T1D-DN1. It is well known that serum creatinine is the most commonly used indicator of renal function [3]. However, serum creatinine levels are influenced by muscle mass, age, medication, and the patient’s diet, which may fail proper renal function evaluation based on [10,36]. Therefore, the use of a single creatinine is not often effective for the diagnosis of DN, but in combination with other biomarkers, it enables better discrimination of patients from individuals without disease, as shown in our study. 

Five out of the revealed amino acids and derivatives (creatinine, L-arginine, L-proline, citrulline, and creatine) with an up-regulation profile in the DN subjects are involved in the urea cycle/amino group metabolism. In our study, serum urea levels as well as serum creatinine were higher in the DN subjects compared with the T1D patients without DN and were much higher in the patients with late-stage than with early. The kidneys eliminate up to 90% of nitrogen from the human body through urine and thus have an effect on the urea cycle and the conversion of ammonia into uric acid and urea [37]. Recently it was shown that serum urea and uric acid are associated with decreased GFR as well as albuminuria and can point to the DN progression [38,39]. Thus, the changes in the metabolites of the urea cycle/amino group metabolism may be used as a warning sign for DN development. 

Among the revealed metabolites with potential prediction significance, there were the following chemical classes of compounds involved in amino acid metabolism: 4-guanidinobutanamide with N2-succinyl-L-ornithine—in arginine and proline metabolism, monosaccharide N-acetyl-b-glucosaminylamine with N-formyl-L-aspartate—in aspartate and asparagine metabolism, and 1-methylhistidine—in histidine metabolism [27]. These results once again suggest that amino acid metabolism plays a key role in DN progression in T1D patients. It was earlier reported that 1-methylhistidine is associated with chronic kidney disease progression and may be linked to the pathogenicity of diabetic endothelial dysfunction in DN [40,41]. Oxalosuccinic acid involved in all the predictive models may point to dysregulation in the citrate cycle (TCA cycle), whose possible role in the pathogenesis of DN and other kidney diseases is reported numerously [17,18]. Metabolic links between the revealed various amino acids metabolism pathways, urea cycle, and citrate cycle make data interpretation complex, and the detailed mechanisms of these pathways in T1D DN need further research.

In addition, one of the metabolites characterized by a significant difference between the T1D patients with and without DN was 3-carboxy-4-methyl-5-propyl-2-furanpropionic acid (CMPF). This metabolite included in all the determined diagnostic signatures is well known as an uremic toxin, which significantly accumulated in the blood of patients with chronic kidney disease [42,43,44,45]. Various uremic toxins were found in the metabolomics studies of DN in type 1 diabetes individuals and can be used for both prediction and management of the disease [16,46].

We have not analyzed for gender differences in the dysregulated metabolites because of the small size of the studied groups, but the reviews including a large number of studies on gender-related differences in DN in T1D populations were published [47,48]. It should be noted that direct dependence between sex and DN risk of development or progression in T1D individuals has not been found to date. It has been reported that men were at a higher prevalence of diabetic kidney disease, while women were at a higher risk of disease progression [49]. The gender difference in the severity of diabetes and diabetic nephropathy was evident with age in the studied cohorts, indicating a key role of sex hormones. It has been shown that the risk of chronic kidney disease development is equal in men and women if T1D was diagnosed during childhood, while sex differences were found if diabetes developed in the peripubertal period. In addition, sex differences are often dependent on the duration of diabetes. For example, the prevalence of DN is higher in men with a T1D duration of 25 years or more [50,51]. The various studies can report heterogeneous results that may be explained by differences in the studied cohorts such as age, diabetes duration, age of diabetes onset, and presence of other comorbidities or risk factors for DN progression, as well as differences in the study design, including the principles of group formation and even the equations used for GFR calculation [48]. That is why most metabolomics studies of DN have not found any gender differences due to their relatively small number of samples or did not perform this analysis at all.

Thus, all the metabolites revealed in our study have shown a distinct association with the biochemical processes related to the pathogenesis and progression of DN, and each of them can be separately considered as a potential biomarker for diagnosing DN. The fact that these metabolites were detected in a number of other metabolomics studies of DN [17,18,38,40] may be considered one of the ways that validate our results. However, very often, using combinations of biomarkers (signatures) provides better discrimination of patients from healthy individuals than using single biomarkers [52,53,54]. In our work, the various combinations of the detected metabolites have shown high diagnostic performance for the early and late stages of the disease. The selected metabolite combinations have shown high performance for diagnosing DN, especially at the late stage (88% and 99%, respectively). The metabolite signature determined for the early stage of DN (the T1D-DN1 group) is characterized by a diagnostic performance of 81%, which can be considered an excellent test. Taking into account that creatinine level was not so high at the early stage of the disease while typical morphological changes in the kidney could be happening already, its combination with other metabolites allowed to enhance the power for early diagnostics, which could provide an opportunity to delay or prevent the disease development by on-time therapy. In addition, the involved metabolites might be useful in the evaluation of the treatment of the disease, which could result in the delay or prevention of DN progression and reduce the risk of renal failure development. 

Some limitations of our study should be considered. First, the sample size was relatively small, especially for patients with late stages of DN; therefore, we could not sufficiently determine the dysregulated metabolites and associated pathways at different stages of DN in the patients with T1D and analyze gender differences in the dysregulated metabolites. Second, as in any cross-sectional study, the causal relationship between T1D, DN, and metabolite changes could not be determined and mechanisms of disease development could be suggested only. For the same reason, the obtained results cannot be applied to predict the DN stage-to-stage progression risk, although some revealed metabolites are characterized by different dysregulation patterns at the early and late stages of the disease. The detection of predictive biomarkers requires a longitudinal study. Therefore, further investigations with huge cohorts, including the prospective longitudinal study, are needed to validate the proposed diagnostic metabolite signatures and determine the relationship between DN progression and the metabolites.

## 5. Conclusions

The untargeted metabolomics analysis coupled with functional analysis based on system biology knowledge allowed us to identify the best combinations of 15 dysregulated metabolites, including amino acids and derivatives, monosaccharides, organic acids, and uremic toxin for DN diagnosis. The selected metabolite combinations have shown high performance in diagnosing DN, especially in the late stage (up to 99%). Despite the metabolite signature determined for the early stage of DN being characterized by a diagnostic performance of 81%, these metabolites as potential biomarkers might be useful in the evaluation of treatment of the disease, especially at early stages that may reduce the risk of kidney failure development than existing creatinine/albumin-based tests.

## Figures and Tables

**Table 1:** Characteristics of the studied cohorts.

	T1D n = 50	T1D-DN1 n = 19	T1D-DN2 n = 11	*p*-Value
Age (years)	31.7 ± 10	47.7 ± 13.8	36.1 ± 9.6	<0.001
Sex (M/F)	26/24	6/13	3/8	
BMI (kg/m^2^)	24.5 ± 5	26.3 ± 4.4	23.4 ± 5.5	0.2213
Years since T1D diagnosed	14.0 ± 8.5	27.5 ± 10.4	25.0 ± 7	<0.001
Systolic blood pressure (mmHg)	121.4 ± 10.7	128.0 ± 12.5	133.2 ± 27.2	0.02863
Diastolic blood pressure (mmHg)	76.5 ± 8.8	79.7 ± 9.2	79.1 ± 11.4	0.38071
HbA1c (%)	8.3 ± 1.5	8.8 ± 1.3	8.5 ± 1.4	0.44263
Fasting Glucose (mmol/L)	8.2 ± 1.5	7.9 ± 0.9	8.5 ± 1.2	0.43648
Creatinine (µmol/L)	70.6 ± 9.5	87.0 ± 11.4	196.1 ± 75.0	<0.001
Urea (mmol/L)	4.6 ± 1.1	6.0 ± 1.8	11.2 ± 4.6	<0.001
Cholesterol (mmol/L)	4.9 ± 1.2	5.1 ± 1.2	5.5 ± 2.0	0.46141
HDL Cholesterol (mmol/L)	1.4 ± 0.4	1.3 ± 0.5	1.6 ± 0.8	0.20182
LDL Cholesterol (mmol/L)	3.0 ± 1.1	3.2 ± 1.0	3.4 ± 1.3	0.43088
Triglycerides (mmol/L)	1.1 ± 0.7	1.2 ± 0.5	1.1 ± 0.5	0.90265
eGFR (mL/min/1.73 m^2^)	114.3 ± 10.1	77.8 ± 8.5	35.7 ± 14.8	<0.001
ACR (mg/mmol)	1.2 ± 0.7	8.8 ± 8.0	34.1 ± 28.8	0

Data presented as mean ± SD. One-way ANOVA *p*-value < 0.05 is considered as statistically significant. BMI–body mass index; HDL–high density lipoproteins; LDL–low density lipoproteins; eGFR–estimated glomerular filtration rate, ACR–albumin-to-creatinine ratio.

**Table 2:** Top pathways enriched with metabolites characterized by significantly altered abundance in DN, as identified by the functional analysis using MetaboAnalyst.

Top Pathways	Compounds	Significant Hits			
	Hits/Total	T1D vs. T1D-DN1	T1D vs. T1D-DN2	T1D-DN1 vs. T1D-DN2	Identified Metabolites
Aspartate and asparagine metabolism	20/114	8	8	5	L-arginine, L-cysteine, 2-ketobutyric acid, L-proline, citrulline, N-formyl-L-aspartate, 4-guanidinobutanamide, N2-succinyl-L-ornithine
Arginine and proline metabolism	12/45	4	4	2	L-arginine, L-proline, citrulline, 4-guanidinobutanamide
Methionine and cysteine metabolism	14/94	4	2	4	L-cysteine, thiosulfate, thiocysteine, 3-sulfopyruvic acid, 5-L-glutamyl-taurine
Alanine and aspartate metabolism	4/30	2	3	1	L-arginine, citrulline, 2-oxosuccinamic acid
Urea cycle/amino group metabolism	12/85	3	5	4	L-arginine, L-proline, creatine, citrulline, creatinine
Glycine, serine, alanine, and threonine metabolism	17/88	2	5	4	L-arginine, creatine, phosphoglycolic acid, L-2-amino-3-oxobutanoic acid, 2-oxo-3-hydroxy-4-phosphobutanoic acid

**Table 3:** The best combinations of metabolites for diagnosing DN cases in the patients with T1D.

	T1D vs. T1D-DN1/DN2	T1D vs. T1D-DN1	T1D vs. T1D-DN2
Creatinine	+	+	+
L-Proline	+	+	+
L-Cysteine	+	+	+
Thiocysteine	−	−	+
4-Guanidinobutanamide	−	−	+
Creatine	+	−	+
N-Formyl-L-aspartate	−	+	−
1-Methylhistidine	+	+	+
2-Oxo-3-hydroxy- 4-phosphobutanoic acid	−	−	+
L-Arginine	+	−	−
Citrulline	+	−	+
Oxalosuccinic acid	+	+	+
N-Acetyl-b-glucosaminylamine	+	+	−
N2-Succinyl-L-ornithine	+	+	+
3-Carboxy-4-methyl-5-propyl- 2-furanpropionic acid	+	+	+

+/− shows the presence/absence of the metabolite in a combination.

## Data Availability

The data presented in this study are available on request from the corresponding author.

## Supplementary Materials

The following supporting information can be downloaded at: https://www.mdpi.com/article/10.3390/jpm12111889/s1, Figure S1: The relative abundance of the altered metabolites in the blood plasma samples of the patients in the T1D, T1D-DN1, and T1D-DN2 groups; Table S1: The annotated metabolites associated with top dysregulated metabolite pathways between the T1D, T1D-DN1, and T1D-DN2 subjects.
