# Supplementary material for: Potential Plasma Metabolite Biomarkers of Diabetic Nephropathy: Untargeted Metabolomics Study
Source: J Pers Med. 2022 Nov 11;12(11):1889. doi: 10.3390/jpm12111889 (PMC9692474; doi:10.3390/jpm12111889)
Supplement: Supplementary file 1 [file jpm-12-01889-s001.zip › jpm-1993179-supplementary.pdf]

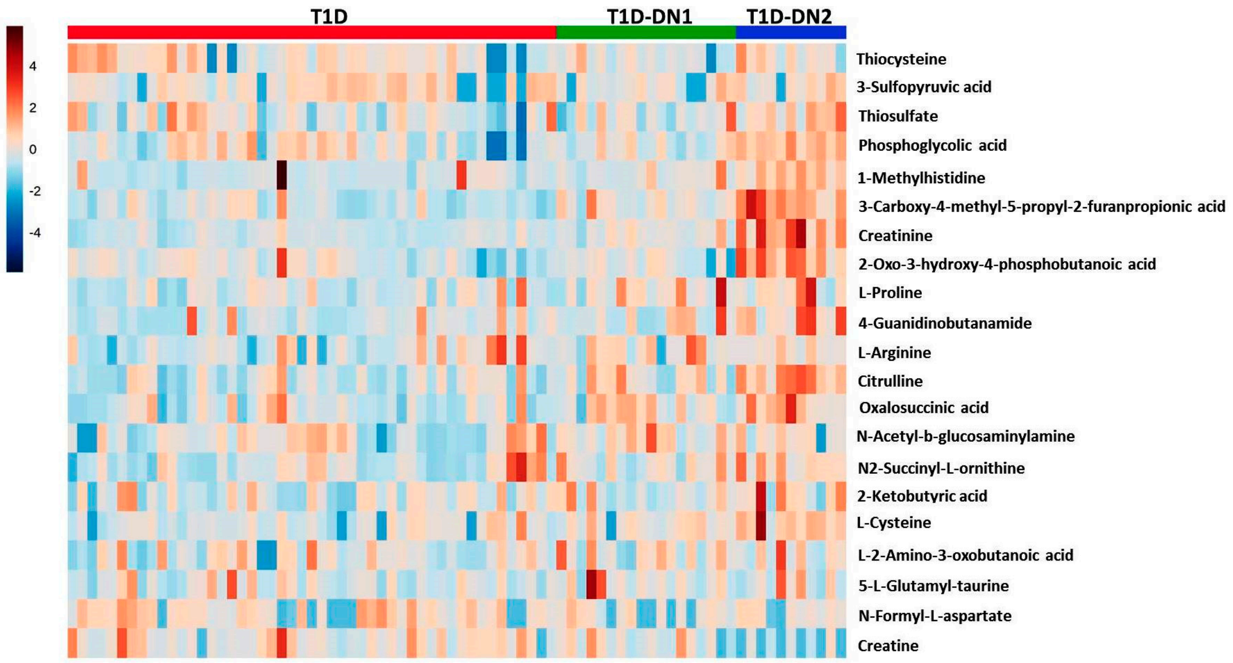

Figure S1. The relative abundance of the altered metabolites in the blood plasma samples of the patients in the T1D, T1D-DN1 and T1D-DN2 groups. The color bar shows the abundance z-scores.

Table S1. The annotated metabolites associated with top dysregulated metabolite pathways between the T1D, T1D-DN1 and T1D-DN2 subjects.

Name of Metabolites	KEGG ID	Elemental Composition	Ion Form	m/z		T1D vs T1D-DN1/DN2		T1D vs T1D-DN1		T1D vs T1D-DN2	
				Calculated	Measured	P-value	AUC	P-value	AUC	P-value	AUC
2-Ketobutyric acid	C00109	$C_4H_6O_3$	$[M-H_2O+H]^+$	85.0289	85.028	0.038	0.606	0.213	0.562	0.012	0.683
4-Guanidinobutanoic acid	C01035	$C_5H_{11}N_3O_2$	$[M-H_4O_2+H]^+$	110.0718	110.071	0.717	0.506	0.537	0.503	0.802	0.513
Creatinine	C00791	$C_4H_7N_3O$	$[M+H]^+$	114.0662	114.065	3.01×10^{-7}	0.860	2.65×10^{-4}	0.785	1.42×10^{-14}	0.988
L-Proline*	C00148	$C_5H_9NO_2$	$[M+H]^+$	116.0712	116.070	0.018	0.679	0.114	0.658	0.0262	0.716
L-Cysteine	C00097	$C_3H_7NO_2S$	$[M+H]^+$	122.0276	122.027	5.24×10^{-4}	0.766	0.042	0.694	1.92×10^{-5}	0.892
2-Ketobutyric acid	C00109	$C_4H_6O_3$	$[M+Na]^+$	125.0215	125.021	0.604	0.548	0.817	0.516	0.483	0.603
4-Hydroxyproline	C01157	$C_5H_9NO_3$	$[M+H]^+$	132.0661	132.065	0.598	0.503	0.143	0.596	0.287	0.658
Creatine*	C00300	$C_4H_9N_3O_2$	$[M+H]^+$	132.0773	132.076	0.725	0.599	0.692	0.540	0.147	0.702
Thiosulfate	C00320	HS_2O_3	$[M+Na]^+$	135.9265	135.926	0.476	0.552	0.535	0.548	0.019	0.725
Thiocysteine	C01962	$C_3H_7NO_2S_2$	$[M-H_2O+H]^+$	135.9891	135.989	0.405	0.630	0.128	0.714	0.580	0.516
Creatinine	C00791	$C_4H_7N_3O$	$[M+Na]^+$	136.0481	136.047	1.37×10^{-5}	0.782	7.89×10^{-3}	0.693	4.96×10^{-9}	0.936
L-Proline*	C00148	$C_5H_9NO_2$	$[M+Na]^+$	138.0531	138.052	1.62×10^{-3}	0.713	6.96×10^{-3}	0.709	6.36×10^{-3}	0.721
Phosphoglycolic acid	C00988	$C_2H_5O_6P$	$[M-H_2O+H]^+$	138.9796	138.979	0.079	0.619	0.863	0.532	3.35×10^{-4}	0.880
L-2-Amino-3-oxobutanoic acid	C03508	$C_4H_7NO_3$	$[M+Na]^+$	140.0324	140.032	0.025	0.626	0.029	0.634	0.175	0.611
4-Guanidinobutanamide	C03078	$C_5H_{12}N_4O$	$[M]^+$	144.1011	144.101	0.013	0.664	0.210	0.558	1.10×10^{-3}	0.847
L-Lysine*	C00047	$C_6H_{14}N_2O_2$	$[M+H]^+$	147.1134	147.113	0.885	0.522	0.665	0.544	0.339	0.516
Phosphoserine	C01005	$C_3H_8NO_6P$	[M-	149.9956	149.995	0.292	0.638	0.412	0.615	0.392	0.679

			$H_2O+H]^+$								
L-Methionine*	C00073	$C_5H_{11}NO_2S$	$[M+H]^+$	150.0589	150.058	0.676	0.525	0.683	0.540	0.170	0.636
Creatinine	C00791	$C_4H_7N_3O$	$[M+K]^+$	152.0221	152.022	3.15×10^{-3}	0.695	0.422	0.579	1.67×10^{-6}	0.896
Creatine*	C00300	$C_4H_9N_3O_2$	$[M+Na]^+$	154.0592	154.058	0.730	0.520	0.410	0.523	0.433	0.515
N-Formyl-L-aspartate	C01044	$C_5H_7NO_5$	$[M]^+$	161.0324	161.033	0.039	0.647	0.036	0.669	0.381	0.610
3-Sulfopyruvic acid	C05528	$C_3H_4O_6S$	$[M+H]^+$	168.9807	168.981	0.818	0.557	0.430	0.611	0.500	0.536
2-Oxosuccinamic acid	C02362	$C_4H_5NO_4$	$[M+K]^+$	169.9856	169.985	0.786	0.603	0.810	0.543	0.426	0.706
Creatine*	C00300	$C_4H_9N_3O_2$	$[M+K]^+$	170.0332	170.032	0.097	0.705	0.949	0.616	1.98×10^{-5}	0.857
1-Methylhistidine	C01152	$C_7H_{11}N_3O_2$	$[M+H]^+$	170.0930	170.092	0.017	0.796	0.291	0.719	3.01×10^{-3}	0.928
3-Sulfinylpyruvic acid	C05527	$C_3H_4O_5S$	$[M+Na]^+$	174.9677	174.967	0.965	0.521	0.591	0.554	0.491	0.536
2-Oxo-3-hydroxy-4-phosphobutanoic acid	C06054	$C_4H_7O_8P$	$[M-H_4O_2+H]^+$	178.9745	178.974	0.022	0.659	0.440	0.507	2.63×10^{-8}	0.946
Oxoglutaric acid	C00026	$C_5H_6O_5$	$[M+K]^+$	184.9852	184.985	0.554	0.545	0.277	0.593	0.623	0.538
Cysteic acid	C00506	$C_3H_7NO_5S$	$[M+H_2O+H]^+$	188.0229	188.022	0.654	0.543	0.361	0.585	0.625	0.528
L-Arginine*	C00062	$C_6H_{14}N_4O_2$	$[M+Na]^+$	197.1014	197.100	0.095	0.672	0.129	0.655	0.356	0.702
Citrulline	C00327	$C_6H_{13}N_3O_3$	$[M+Na]^+$	198.0855	198.084	2.29×10^{-5}	0.750	0.025	0.646	1.18×10^{-8}	0.928
Phosphoserine	C01005	$C_3H_8NO_6P$	$[M+H_2O+H]^+$	204.0273	204.026	0.363	0.545	0.647	0.527	0.295	0.574
3-Sulfopyruvic acid	C05528	$C_3H_4O_6S$	$[M+K]^+$	206.9366	206.937	0.648	0.527	0.986	0.511	0.407	0.594
Oxalosuccinic acid	C05379	$C_6H_6O_7$	$[M+Na]^+$	213.0011	212.999	1.31×10^{-4}	0.761	5.85×10^{-3}	0.717	1.10×10^{-4}	0.838
L-Arginine*	C00062	$C_6H_{14}N_4O_2$	$[M+K]^+$	213.0754	213.074	0.496	0.548	0.555	0.522	0.658	0.594
Cysteinylglycine	C01419	$C_5H_{10}N_2O_3S$	$[M+K]^+$	217.0049	217.005	0.201	0.534	0.165	0.555	0.576	0.503
N-Acetyl-b-glucosaminylamine	C01239	$C_8H_{16}N_2O_5$	$[M+H]^+$	221.1137	221.114	0.196	0.635	0.268	0.624	0.432	0.656

DL-Homocystine	C01817	$C_8H_{16}N_2O_4S_2$	$[M-H_4O_2+H]^+$	233.0418	233.041	0.778	0.571	0.308	0.620	0.294	0.513
N-Acetyl-b-glucosaminylamine	C01239	$C_8H_{16}N_2O_5$	$[M+H_2O+H]^+$	239.1243	239.124	0.018	0.676	0.010	0.699	0.384	0.637
Lipoamide	C00248	$C_8H_{15}NOS_2$	$[M+K]^+$	244.0232	244.023	0.772	0.538	0.879	0.531	0.436	0.551
N2-Succinyl-L-ornithine	C03415	$C_9H_{16}N_2O_5$	$[M+H_2O+H]^+$	251.1243	251.125	4.64×10^{-3}	0.727	0.079	0.673	4.16×10^{-3}	0.820
3-Carboxy-4-methyl-5-propyl-2-furanpropionic acid	NA	$C_{12}H_{16}O_5$	$[M+Na]^+$	263.0895	263.085	1.71×10^{-7}	0.842	1.06×10^{-3}	0.76932	4.15×10^{-12}	0.967
5-L-Glutamyl-taurine	C05844	$C_7H_{14}N_2O_6S$	$[M+K]^+$	293.0210	293.021	0.076	0.593	0.059	0.625	0.369	0.538

P-value obtained from the pairwise Wilcoxon rank-sum test; AUC - area under curve, obtained ROC curve based model evaluation; * - metabolites identified by MS/MS fragmentation.
